# Copper(II) complexes with imino phenoxide ligands: synthesis, characterization, and their application as catalysts for the ring-opening polymerization of *rac*-lactide

**DOI:** 10.1007/s00706-016-1830-7

**Published:** 2016-09-05

**Authors:** Mrinmay Mandal, Kerstin Oppelt, Manuela List, Ian Teasdale, Debashis Chakraborty, Uwe Monkowius

**Affiliations:** 1 Institute of Inorganic Chemistry, Johannes Kepler University Linz, Altenbergerstr. 69, 4040 Linz, Austria; 2 Department of Chemistry, Indian Institute of Technology Patna, Patna, Bihar 800 013 India; 3 Institute for Chemical Technology of Organic Materials, Johannes Kepler University Linz, Altenbergerstr. 69, 4040 Linz, Austria; 4 Institute of Polymer Chemistry, Johannes Kepler University Linz, Altenbergerstr. 69, 4040 Linz, Austria; 5 Department of Chemistry, Indian Institute of Technology Madras, Chennai, Tamil Nadu 600 036 India

**Keywords:** Imino phenoxide, Copper, Crystal structure, ROP, *rac*-Lactide, Cyclic voltammetry, Poly(lactic acid)

## Abstract

**Abstract:**

Four new copper complexes based on bidentate imino phenoxide ligands were synthesized and characterized by IR, UV–Vis spectroscopy, ESI mass spectrometry, single crystal X-ray diffraction, and electrochemistry. The crystal structures revealed that the copper(II) atoms are surrounded by phenolate oxygen and imine nitrogen atoms of two ligands in a distorted square-planar geometry. The existence of ligand-centered, as well as Cu(II)-centered quasi-reversible and reversible redox reactions are observed in the cyclic voltammetry experiments of all the complexes. All complexes are able to catalyze the ring-opening polymerization of *rac*-lactide yielding polymers with moderate molecular weights and moderately broad molecular weight distributions.

**Graphical abstract:**

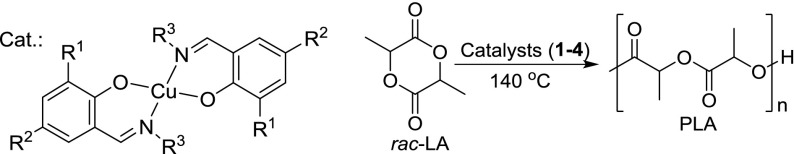

**Electronic supplementary material:**

The online version of this article (doi:10.1007/s00706-016-1830-7) contains supplementary material, which is available to authorized users.

## Introduction

In a similar fashion to tetradentate salen-type ligands, the related bidentate imino phenoxide ligands have proven to be extremely versatile ligands for main group, as well as transition metals. Such ligands have been used in almost all areas of coordination chemistry to prepare complexes which are catalytic and biological active or which feature interesting structural, electrochemical or magnetic properties [1, 2].

In recent studies, we have used imino phenoxide ligands to prepare early transition and main group metal complexes as polymerization catalysts, in particular, for the preparation of aliphatic polyesters such as poly(lactic acid) (PLA) and poly(caprolactone) (PCL) [3–8]. Such aliphatic polyesters are interesting, as one of the most important families of environmentally benign biodegradable polymers [9–14]. The degradation pathway is well studied: PLA degrades to the metabolisable lactic acid [15–17]. Generally, aliphatic polyesters are synthesized by the ring-opening polymerization (ROP) of cyclic esters using metal-based initiators [18–25]. Sn(Oct)_2_ is traditionally used for the industrial production of PLA, however, it is not suitable for producing PLA for biomedical applications due to the toxicity of the residual metal [13, 26]. Therefore, the syntheses of complexes with biologically benign metals are important.

Cu(II)-based catalysts have been widely investigated for the ROP of lactide (LA) before [27–33]. All studied catalytic systems are able to polymerize LA, however, with low activity and selectivity and at high temperatures. More recently, Cu(II) complexes bearing either the ligand set diketiminate/isopropoxide or ligands based on cyclohexane-1,2-diamine were used as catalysts for the polymerization of *rac*-lactide (*rac*-LA) [34, 35]. The catalysis of these systems was very efficient resulting in the formation of PLA in a controlled manner and displayed very high activities. Also diimino pyrrolide copper alkoxide complexes for the isotactic ROP of *rac*-LA gives polymers with suitably high *M*_n_ (44–45 kg/mol) and controlled molecular weight distributions (MWDs) (1.0–1.2) [36]. On the other hand, Wang et al. reported copper complexes bearing bis(imino) phenoxide derived ligands with reactivities which were practically either zero or the formation of only trace polymer was observed for the ROP of ε-caprolactone [37].

In continuation of our previous work on metal complexes containing imino phenoxide ligands, we describe herein the use of these ligands for the synthesis of four novel Cu(II) complexes. In principle, this class of complexes is well studied for different applications [38–55]. These complexes are easy to prepare and stable towards air and moisture and, therefore, they are much easier to handle compared to the sensitive polymerization catalysts based on 1st, 4th, 5th, and 13th group metals [3–7]. The imino phenoxide ligands used in the study were available from previous studies. Due to the fact that these Cu(II) complexes feature interesting structural, electronic, and spectroscopic properties, we also studied these compounds by single crystal X-ray diffraction, UV–Vis spectroscopy, and spectro-electrochemistry. Finally, their catalytic efficiency towards the polymerization of *rac*-LA was investigated.

## Results and discussion

### Synthesis and characterisation

The bidentate imino phenoxide ligands **L1**–**L4** were prepared following a procedure reported in the literature [56, 57]: the complexes were synthesized by mixing a methanolic solution of the Schiff base and copper acetate (0.5 mmol) in 10 cm^3^ methanol. After refluxing for 2 h the complexes **1**–**4** could be isolated in high yields and purities (Scheme 1).

**Figure:**
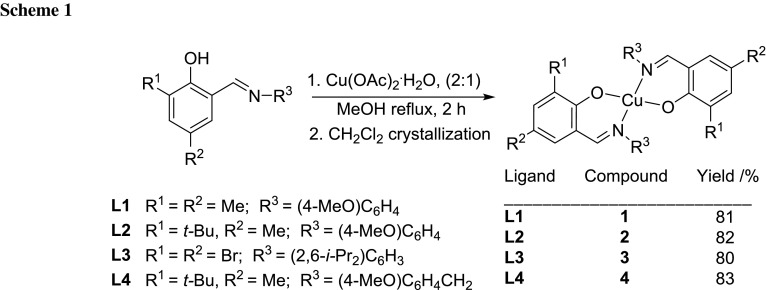

The purity of the complexes was confirmed by elemental analysis. Electrospray ionization mass spectra show the presence of L_2_CuH^+^/L_2_CuNa^+^ ion peaks indicative for a 2:1 structure. The coordination of ligands to the copper(II) atom is also visible in the IR spectra (Figs. S1 and S2): the shift of C=N stretching frequency of the ligands (1623–1632 cm^−1^) towards lower frequencies (1606–1620 cm^−1^) upon complexation confirmed the coordination of the azomethine nitrogen to the metal atom. The signals in the ^1^H NMR spectra of all complexes are very broad due to the paramagnetic nature of the Cu(II) ion and hence, no further NMR analysis was performed.

### Structural studies

**Fig. 1:**
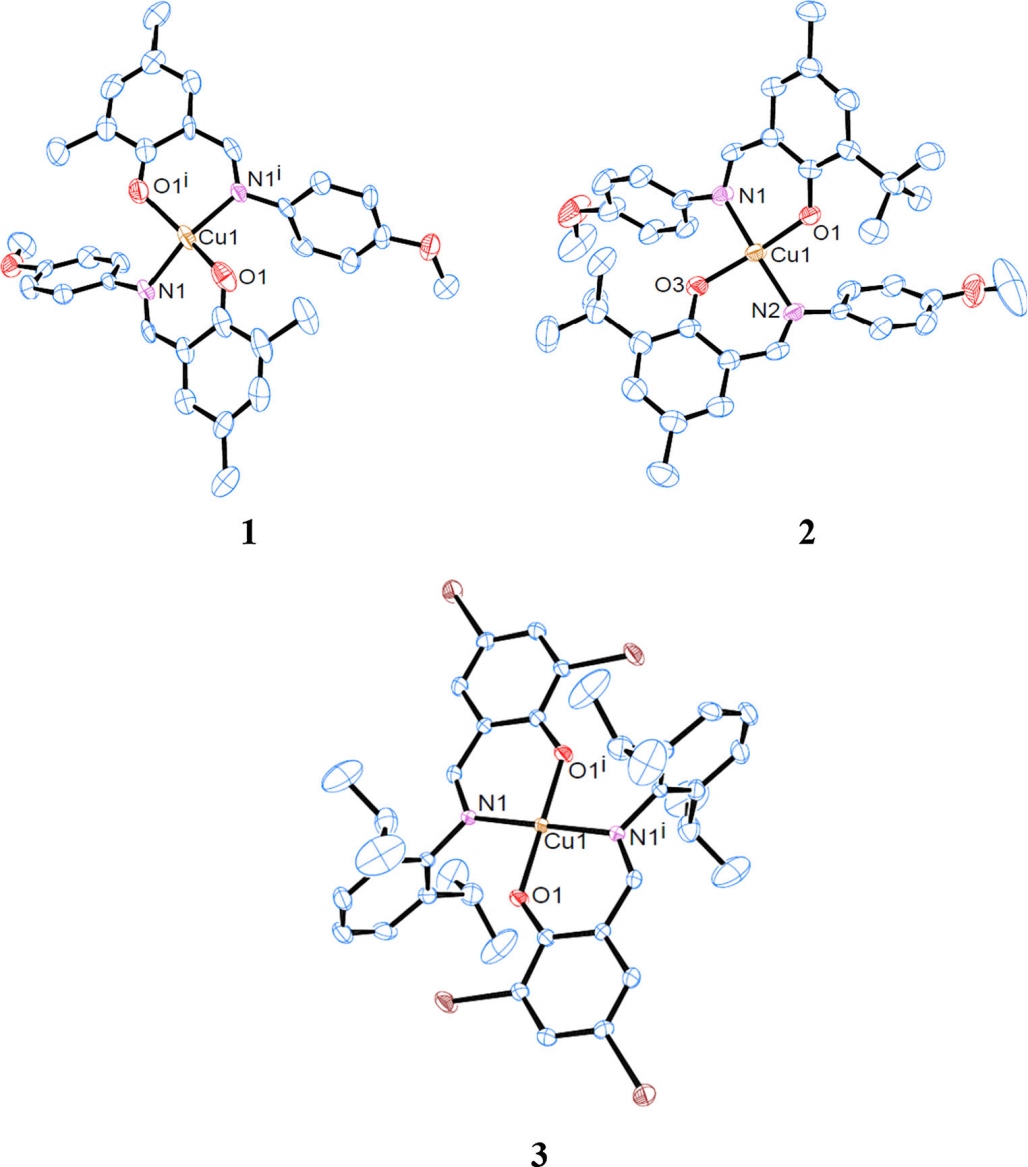
Molecular structures of **1**–**3**. Displacement ellipsoids were drawn at 50 % probability level (exception **1**: 30 %). Hydrogen atoms are omitted for clarity

All complexes were obtained as crystalline solids suitable for single crystal X-ray analysis by slow evaporation of dichloromethane from their solutions. Because crystals of **4** were of very low quality also the resulting crystallographic data are very poor. Therefore, these data are not discussed here but can be found in the ESI for the sake of completeness (Fig. S3 and Table S1). Detailed crystallographic data for **1**–**3** are presented in Table 5. The molecular structures of **1**–**3** are depicted in Fig. 1 and selected bond lengths and bond angles are summarized in Table 1. Complex **1** was found to crystallize in the orthorhombic space group *Fdd*2, complex **3** in the triclinic space group *P* $\bar{1}$. For both complexes, the asymmetric unit consist of one half of a formula unit. Complex **2** crystallizes in the monoclinic space group *C*2/*c*, complex **4** in *P* $\bar{1}$ with each containing one formula unit per asymmetric unit. In all complexes, the copper atom is coordinated by two phenolate oxygen and two imine nitrogen atoms of the ligands in a *trans* configuration. For **3**, the copper atom exists in a perfect square-planar coordination environment. In the other complexes the coordination geometry deviates considerably from planarity due to steric repulsion of the alkyl-substituents in *ortho* position of the phenoxide moiety. For **3**, the bromine atom is somewhat smaller than the alkyl groups, which reduces the steric hindrance. Additional stabilization is gained by C–Br/π-interactions: the bromine atom points directly to the π-system of the phenyl group of the aniline moiety with a distance between the ring plane and the bromine atom of ~3.38 Å which is typical for such interactions [38, 39]. In the complexes, the Cu–O bond distances are in average only slightly shorter than the Cu–N bonds (~1.92 vs. ~1.97 Å). The bite angle of the ligand in **3** is considerably smaller (87.95°) than the bite angle of the other three complexes (92.1°–93.3°). These structural parameters are very similar to reported examples of this group of complexes [38–51]. For all complexes, the crystal packing is stabilized by multiple interactions like CH/π, π/π, CH/O, and C(benzylic)/π interaction (see crystal packing diagrams in Figs. S4–S6, ESI). The structural parameters for these weak interactions are comparable with literature values [58–61].

**Table 1:** Selected bond lengths/Å and bond angles/° for **1**–**3**

	**1**	**2**	**3**
Cu–O	1.921 (10)	1.899 (3) 1.889 (3)	1.896 (3)
Cu–N	1.968 (9)	1.982 (4) 1.972 (4)	1.990 (3)
O–Cu–O	152.4 (7)	156.33 (17)	179.999 (1)
N–Cu–N	153.5 (6)	161.73 (19)	180.0
O–Cu–N^a^ O–Cu–N	93.1 (4) 93.1 (4)	92.32 (16)/92.03 (15) 91.46 (15)/91.66 (16)	92.05 (13) 87.95 (13)

^a^Bite angle of the ligand

### Electronic spectra

**Fig. 2:**
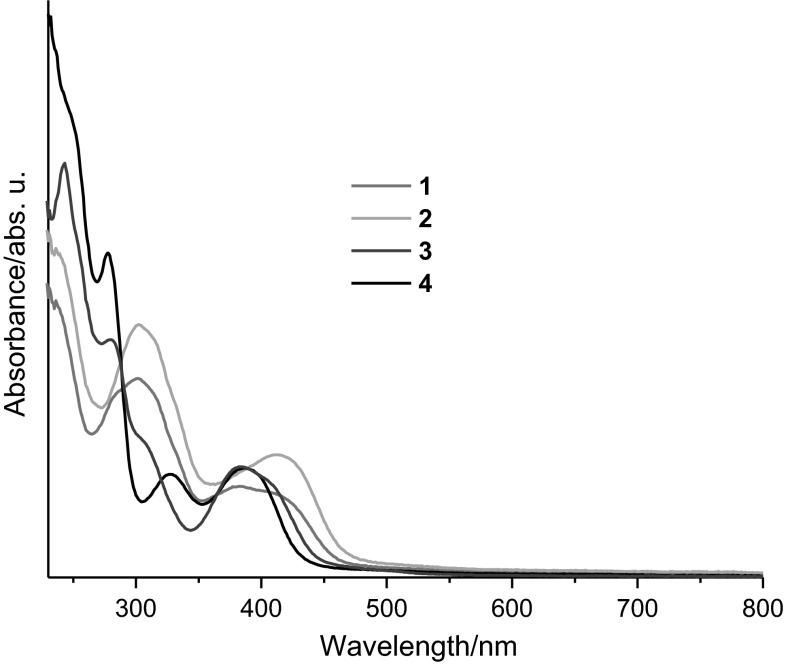
UV–Vis absorption spectra of **1**–**4** in dichloromethane (*c* ≈ 10^−5^ mol dm^−3^)

The electronic spectra of all complexes were recorded in CH_2_Cl_2_ in the range of 200–800 nm. The data are summarized in Table 2. The bands below 350 nm are assigned to ligand π–π* transitions (Fig. 2) [62]. The broad bands between 380 and 410 nm are attributed to ligand-to-metal charge transfers (LMCT) [44]. For **2**–**4** very weak d–d transitions are found beyond 650 nm at very high concentrations (Fig. S7) [46, 53].

**Table 2:** UV–Vis spectroscopic data of the complexes **1**–**4**

Substance	*λ*/nm (*ε*/log(dm^3^ mol^−1^ cm^−1^))
**1**	281 (sh, 4.40), 302 (4.44), 315 (sh, 4.40), 381 (4.10), 410 (4.07)
**2**	301 (4.46), 314 (4.43), 413 (4.15), 681
**3**	243 (4.68), 280 (4.44), 306 (sh, 4.19), 384 (4.11), 408 (sh, 4.10), 661
**4**	245 (sh, 4.46), 277 (4.30), 328 (3.80), 387 (3.83), 757

### (Spectro)electrochemistry

The electrochemical behavior of complexes **1**–**4**, as well as of ligand **L3** was investigated by cyclic voltammetry (CV) in CH_2_Cl_2_ solutions containing 0.1 M of [Bu_4_N]PF_6_ as supporting electrolyte. The ferrocene/ferrocenium (Fc/Fc^+^) redox pair was used as internal standard. The oxidative and the reductive scan were conducted separately due to the deposition of a brownish-red metallic material (presumably copper) on the working electrode which could be observed after cathodic scanning.

**Fig. 3:**
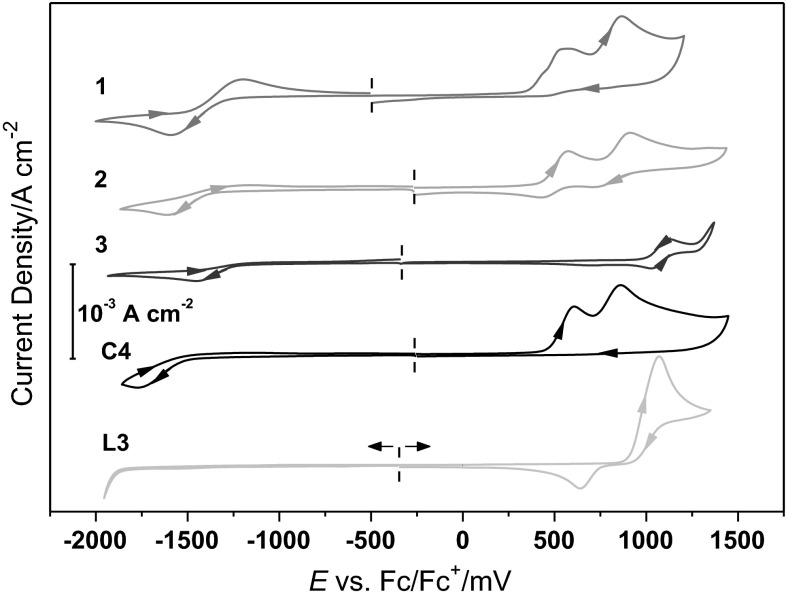
Cyclic voltammograms of complexes **1**–**4** and ligand **3**

Within the electrochemical potential window of the solvent and electrolyte, all investigated complexes show a single reduction (Fig. 3). Most authors assign this first reduction potential to the reduction of the neutral Cu(II) imino phenoxide complex to the anionic Cu(I) complex. The stability of the reduced form varies significantly among the complexes and this is represented by large differences of the *I*_pa_/*I*_pc_ ratio of the reductive wave which is a measure of different reversibilities of the redox process. The redox potential for Cu(II) alkyl imino phenoxide shows differences for square-planar and non-planar coordination and has been systematically studied by polarography [52]. The authors found that the higher the deviation from the square-planar coordination the higher the reduction potential, i.e., the easier the complex is reduced. However, no such purely structural-based trends could be observed for the Cu(II) aryl imino phenoxide complexes in our study. The substituents of the ligands seem to have strong electronic effects that influence the redox potential more than the structures. Complex **1** shows a quasi-reversible reduction wave with a half-wave potential of −1391 mV vs. Fc/Fc^+^ (*I*_pa_/*I*_pc_ ~0.9, Table 3). For complex **2**, the oxidative peak of the first reduction potential shows much lower current density, hence the ratio *I*_pa_/*I*_pc_ cannot be determined reliably. This effect has been observed earlier with similar compounds and has been ascribed to the decomposition of the complexes due to the instability of the singly reduced species via a process of ligand loss and immediate further reduction from Cu(I) to Cu(0).

**Table 3:** Electrochemical properties of **1**–**4** measured by cyclic voltammetry; potentials are given against the ferrocene/ferrocenium redox couple in mV Estimated error ±3 %

Catalyst	*E*_red_[Cu^II^L_2_/Cu^I^L_2_^−^] *E*_pc_	*E*_½red_ [Cu^II^L_2_/Cu^I^L_2_^−^] *E*_1/2_	*E*_ox_ [Cu^II^L_2_/Cu^II^L_2_^+^] *E*_a_	*E*_½ox_[Cu^II^L_2_/Cu^II^L_2_^+^] *E*_1/2_
1	–	−1391	546; 869	–
2	–	−1377	–	503; 813
3	−1457	–	–	1077
4	−1778	–	606; 852	–

In a recent publication, the oxidation behavior of similar compounds were ascribed to the formation of a phenoxyl radical [54]. This and further oxidation to a quinine-type oxidation product has been supported by EPR spectroscopy upon chemical oxidation with cerium sulfate for methoxy-substituted imino phenoxide Cu(II) complexes [52]. Complexes **1**, **2**, and **4** show similar electrochemical behaviors as described before with different degrees of reversibility of the oxidation reaction [52, 55]. The oxidation potential of complex **3** is much higher than for the other complexes (Fig. 3). This observation is in line with the assumption that the oxidation is a ligand based process. Contrary to the other ligands, **L3** contains no methyl-substituents but electron-withdrawing bromo-ligands which results in the electron-poorest and hence hardest to oxidize ligand.

**Fig. 4:**
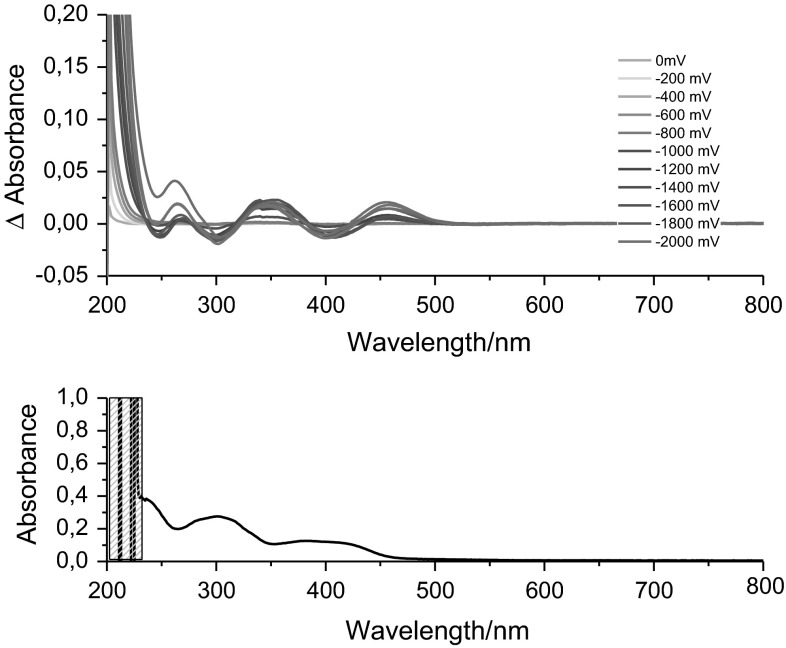
Spectro-electrochemistry of complex **1** in 0.3 M DCE vs. Ag/AgCl (*top*) and UV–Vis spectra of complex **1** in DCM for comparison (*bottom*)

Because it is the only compound featuring a reversible reduction, just **1** was further investigated by spectro-electrochemistry, which was performed in dichloroethane (DCE) solution containing 0.3 M of [Bu_4_N]PF_6_ as supporting electrolyte using an optically transparent thin layer electrochemical (OTTLE) cell (Fig. 4). As reported above, Cu(II) is reduced to Cu(I) under reductive conditions. This results in an change of the nature of lowest-energy transition: Cu(I) as a closed-shell d^10^ ion does not show any d–d or LMCT transitions but metal-to-ligand transition (MLCT) at comparable energies like the LMCTs for Cu(II). Hence, the maximum of the Cu(II) LMCT band at ~400 nm bleaches, whereas two new bands form at ~353 and ~456 nm which are typical for an MLCT of Cu(I) compounds bearing aromatic ligands [63, 64].

### Polymerization studies

All four complexes were able to initiate the ROP of *rac*-LA under solvent free conditions generating PLA of moderate molecular weights (5.30–6.04 kg/mol) and relatively high polymer dispersities (MWDs = 1.71–1.83, Scheme 2). The polymerizations were carried out at 140 °C in a ratio of 200:1 (*rac*-LA:catalyst). The results are summarized in Table 4. The appearances of broader MWDs could be partly explained by transesterification during the polymerization process [65, 66], as well as the slow initiation rates in comparison with a fast propagation. Steric and electronic properties of the ligands affect the catalytic efficiency. Complex **4** is the only one in this series with a benzyl and not an aryl group bound to the imine nitrogen atom, thus rendering the metal center less sterically hindered. Therefore, *rac*-LA can approach readily towards the metal center, thereby appeared to be the best catalyst amongst the complexes investigated in this study. As the activities of all catalysts were found to be very low under bulk condition compared to published reports using copper complexes [34–36], further polymerizations in solution were not carried out. The low activity might be due to the fact that the Cu(II) atoms are coordinatively saturated in the presented complexes. Both accepted polymerization mechanisms, the coordination-insertion and the monomer-activated mechanism, involve the coordination of the carboxylate oxygen atom to the metal atom of the catalyst [67]. Although it is known that Cu(II) is capable of forming five-coordinate square-pyramidal complexes, the tendency to coordinate an additional donor atom of a substrate molecule seems to be low. Furthermore, the complexes seem to be rather stable. Therefore, the formation of a reactive intermediate with a coordinatively unsaturated metal atom by ligand dissociation is unlikely.

**Figure:**
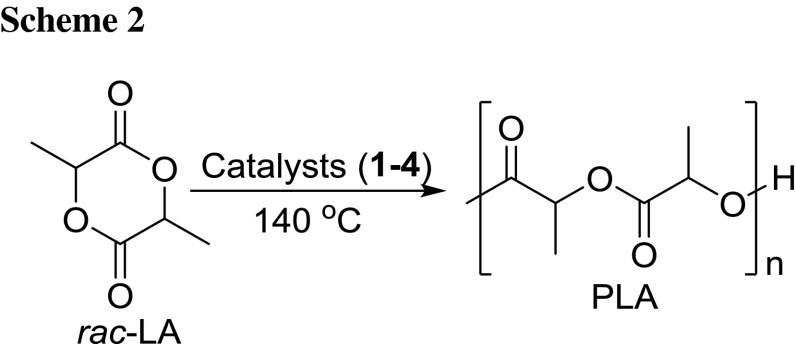

**Table 4:** Polymerization data for *rac*-LA using **1**–**4** in 200:1 ratio (*rac*-LA:catalyst) at 140 °C

Entry	Catalyst	Yield/%	*M*_n_^obs^/kg mol^−1a^	*M*_w_/*M*_n_	*k*_app_/×10^−3^ min^−1b^
1	**1**	94	5.45	1.78	9.361
2	**2**	95	5.76	1.75	10.58
3	**3**	93	5.30	1.83	7.562
4	**4**	95	6.04	1.71	12.78

^a^Measured by GPC at 60 °C in DMF relative to polystyrene standards

^b^As measured from the NMR study

**Fig. 5:**
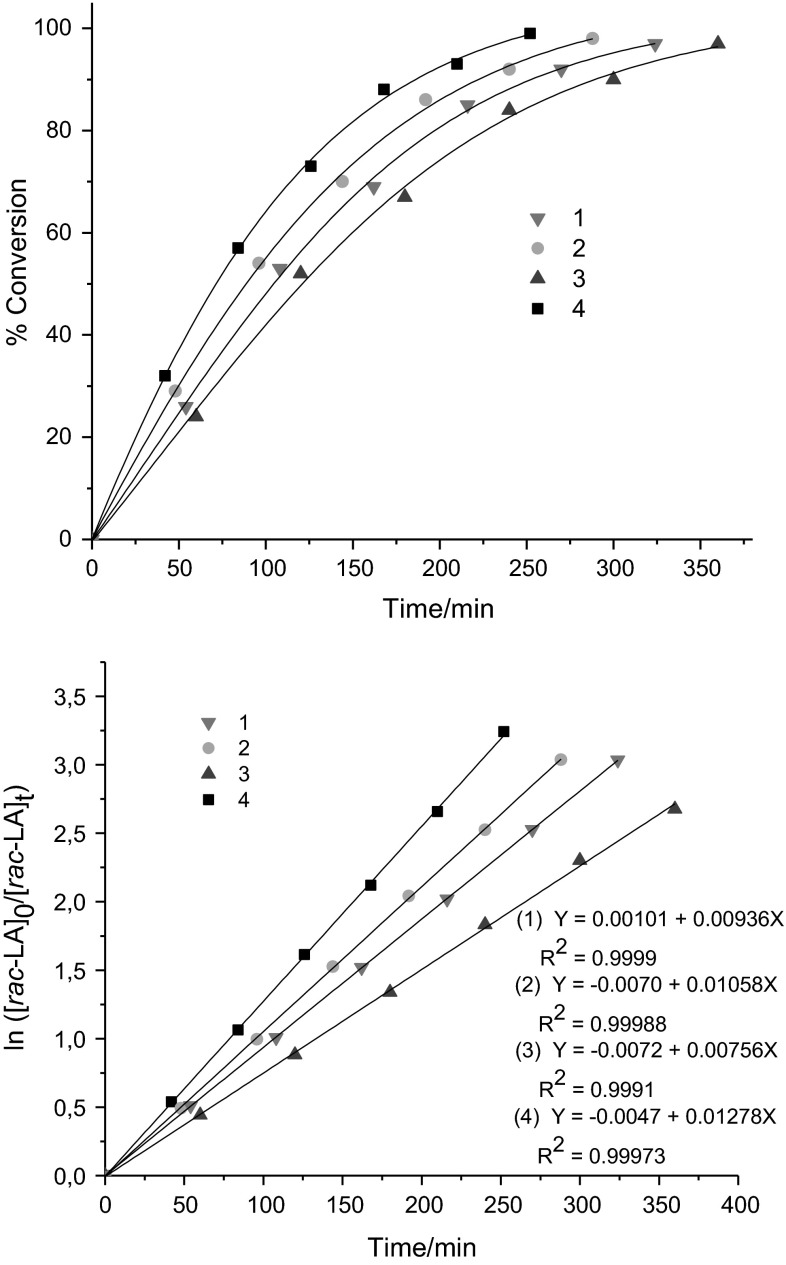
*rac*-LA conversion vs. time (*top*) and ln([LA]_0_/[LA]_*t*_) vs. time plot (*bottom*) using **1**–**4**: [*rac*-LA]_0_:[Cat]_0_ = 200:1 at 140 °C

The polymerization kinetics of *rac*-LA in 200:1 ratio ([*rac*-LA]_0_:[Cat]_0_) using complexes **1**–**4** were investigated by monitoring the reaction via ^1^H NMR spectroscopy. Aliquots of the reaction mixtures were taken out at regular time intervals and the percentage conversion of unreacted monomer to polymer was measured by comparing the peaks at 4.99–5.06 ppm (unreacted monomer) and 5.11–5.25 (polymer). The plot of %-conversion of *rac*-LA against time described a sigmoid curve (Fig. 5, left). The plot of ln([LA]_0_/[LA]_*t*_) vs. time exhibited a good linear relation and indicates that the polymerization proceeds with first order dependence on monomer concentration (Fig. 5, right). The values of the apparent rate constant (*k*_app_) for *rac*-LA polymerization catalyzed by **1**–**4** were evaluated from the slope of these regression lines and are found to be 9.36 × 10^−3^, 10.6 × 10^−3^, 7.56 × 10^−3^, and 12.8 × 10^−3^ min^−1^ for **1**–**4**, respectively. The polymerization rate is fastest for **4** and slowest for **3**.

## Conclusion

In summary, we have synthesized four new copper(II) complexes containing imino phenoxide ligands. These compounds were characterized by spectroscopy (IR, UV–Vis), mass spectrometry, single crystal X-ray crystallography, electrochemistry, and elemental analysis. All complexes feature a metal-based reduction from Cu(II) to Cu(I) and a ligand-centered oxidation. Only for complex **1**, the reduction is quasi-reversible, whereas oxidation of all complexes is irreversible. All complexes exhibited low activity towards the ROP of *rac*-LA in comparison to the literature reported protocols using Cu(II) complexes. The catalytic activity of **4** was found to be highest.

## Experimental

All manipulations were carried out in an atmosphere of dry nitrogen using standard Schlenk techniques. CDCl_3_ used for NMR spectral measurements was dried over calcium hydride for 48 h, distilled and stored in a glove box. IR spectroscopy was performed on a Shimadzu IRAffinity-1 FTIR spectrophotometer that was equipped with a Specac Golden Gate™ single-reflection diamond ATR accessory. Mass spectra were collected on a Finnigan LCQ DecaXP^Plus^ Ion Trap Mass spectrometer with an ESI ion source. Elemental analyses were carried out at the Institute for Chemical Technology of Organic Materials at JKU. For photophysical characterization, spectroscopic grade solvents were used throughout all measurements. Absorption spectra were recorded with a Varian Cary 300 or 50 spectrophotometer. All *ε* values are given in dm^3^ mol^−1^ cm^−1^. Cyclic voltammetry (CV) experiments were carried out under inert conditions in a glovebox using an Eco Autolab potentiostat with a self-made three-electrode cell consisting of a BASi platinum working electrode, a platinum wire as a counter electrode and a silver/silver chloride quasi-reference electrode. The Pt working electrode was polished with 0.1 mm alumina powder and washed with dilute HNO_3_ and double distilled water. The complexes were dissolved in dichloromethane with 0.1 M tetrabutylammonium hexafluorophosphate (TBAPF_6_) as the supporting electrolyte. Scanning rates of 50–200 mV/s were applied. Ferrocene was employed as internal standard for potential referencing, and the potentials were subsequently referenced vs. SCE accordingly [68]. Cyclic voltammograms of all complexes were recorded in the potential range of +1.6 to −1.6 V. Spectro-electrochemistry was performed in a thin layer OTTLE cell [69] with a platinum working electrode, a platinum wire as a counter electrode, and a silver reference electrode. The corresponding UV–Vis absorption spectra were collected with a Jasco V670 Spectrometer.

### Materials

Dichloromethane was dried and distilled over K_2_CO_3_, and methanol was dried and distilled over Na. Cu(OAc)_2_·2H_2_O was purchased from Sigma-Aldrich and used without further purification. *rac*-LA was purchased from Sigma-Aldrich and sublimed under a nitrogen atmosphere repeatedly for further purification and stored in a glove box. All other solvents and reagents were commercially available and used as received. Ligands **L1**–**L4** were prepared according to literature reported procedures [56, 57].

### Synthesis of complexes 1–4

General procedure for the synthesis of **1**–**4**: a methanolic solution (20 cm^3^) of the Schiff base (1 mmol) and copper acetate (0.5 mmol) in 10 cm^3^ methanol were mixed thoroughly and the mixture was heated under reflux for 2 h and then cooled to room temperature. After filtration the resulting solution was evaporated to dryness and the residue was recrystallized from dichloromethane.

#### *Bis[2*-*[(4-methoxyphenyl)iminomethyl]*-*4,6*-*dimethylphenolato-κ*^*2*^*N,O*^*1*^*]copper(II)* (**1**, C_32_H_32_CuN_2_O_4_)

Yield 0.08 g (81 %); ESI–MS: *m/z* calculated for C_32_H_32_CuN_2_O_4_Na ([M+Na]^+^) 594.17, found 594.33; IR (ATR): $\bar{\nu }$ = 1619 (–CH=N), 1506 (Ar–OMe) cm^−1^.

#### *Bis[2*-*[(4-methoxyphenyl)iminomethyl]*-*4*-*methyl*-*6*-*(tert-butyl)phenolato*-*κ*^*2*^*N,O*^*1*^*]copper(II)* (**2**, C_38_H_44_CuN_2_O_4_)

Yield 0.07 g (82 %); ESI–MS: *m/z* calculated for C_38_H_44_CuN_2_O_4_Na ([M+Na]^+^) 678.30, found 678.40; IR (ATR): $\bar{\nu }$ = 1616 (–CH=N), 1506 (Ar–OMe) cm^−1^.

#### *Bis[2,4*-*dibromo*-*6*-*[(2,6*-*diisopropylphenyl)iminomethyl]phenolato*-*κ*^*2*^*N,O*^*1*^*]copper(II)* (**3**, C_38_H_40_Br_4_CuN_2_O_2_)

Yield 0.07 g (80 %); ESI–MS: *m/z* calculated for C_38_H_41_Br_4_CuN_2_O_2_ ([M+H]^+^) 935.93, found 935.13; IR (ATR): $\bar{\nu }$ = 1606 (–CH=N) cm^−1^.

#### *Bis[2*-*[(4*-*methoxybenzyl)iminomethyl]*-*4*-*methyl*-*6*-*(tert*-*butyl)phenolato*-*κ*^*2*^*N,O*^*1*^*]copper(II)* (**4**, C_40_H_48_CuN_2_O_4_)

Yield 0.06 g (83 %); ESI–MS: *m/z* calculated for C_40_H_48_CuN_2_O_4_Na ([M+Na]^+^) 706.29, found 706.33; IR (ATR): $\bar{\nu }$ = 1620 (–CH=N), 1513 (Ar–OMe) cm^−1^.

### X-ray structure determination of complexes 1–4

Suitable single crystals for X-ray diffraction were obtained for all four compounds under ambient conditions from concentrated dichloromethane solution of the respective compounds over a period of 7 days. Diffraction data were collected on a Bruker Smart X2S diffractometer operating with Mo Kα radiation (*λ* = 0.71073 Å). The structures were solved by direct methods (SHELXS-97) [70, 71] and refined by full-matrix least squares on *F* ^2^ (SHELXL-97) [72, 73]. The H atoms were calculated geometrically, and a riding model was applied in the refinement process. These data were deposited with CCDC with the following numbers: CCDC 1448157–1448160 and can be obtained free of charge from the Cambridge Crystallographic Data Centre at https://summary.ccdc.cam.ac.uk/structure-summary-form. Crystal data are given in Table 5 and S1.

**Table 5:** Crystal data for the structures of **1**–**3**

Compound	**1**	**2**	**3**
Empirical formula	C_32_H_32_CuN_2_O_4_	C_38_H_44_CuN_2_O_4_	C_38_H_40_Br_4_CuN_2_O_2_
Formula weight	572.14	656.29	939.90
Crystal system	Orthorhombic	Monoclinic	Triclinic
Space group	*Fdd*2	*C*2*/c*	*P* $\bar{1}$
Temp/K	300	300	300
*a*/Å	34.075 (2)	14.640 (2)	9.3893 (8)
*b*/Å	12.3830 (9)	29.944 (3)	9.4713 (10)
*c*/Å	13.3134 (10)	17.637 (2)	12.4322 (13)
*α*/°	90	90	112.224 (3)
*β*/°	90	114.285 (4)	95.405 (3)
*γ*/°	90	90	99.214 (3)
*V*/Å^3^	5617.6 (7)	7047.5 (16)	995.65 (17)
*Z*	8	8	1
*D*_calc_/g cm^−3^	1.353	1.237	1.568
Reflns collected	17380	63378	18297
Indep. reflns	2016	8153	3517
Obs. reflns [*I* > 2*σ*(*I*)]	1931	3619	2801
Param. refin./restr.	181/1	417/0	218/0
Absorption correction	Multi-scan	Multi-scan	Multi-scan
*R*_1_	0.059	0.060	0.0459
*wR*_2_	0.128	0.17	0.128
CCDC	1448157	1448158	1448159

### General procedure for the bulk polymerization of *rac*-LA

The polymerizations were performed under solvent free conditions in 200:1 ratio of *rac*-LA and the complexes with 173 μmol of **1**–**4** and 5.00 g of *rac*-LA (34.7 mmol) under a nitrogen atmosphere in a 50 cm^3^ flask. Under stirring, the flask was heated to 140 °C. Once the monomer melted fully, a rise in viscosity due to the polymerization reaction was observed and finally the stirring ceased. Then the reaction mixture was dissolved into a minimum quantity of CH_2_Cl_2_ and poured into cold methanol. The polymer precipitated immediately and was collected by filtration. The filtered product was dried in vacuum until a constant weight was achieved. Cold methanol was used for the quenching of the polymerization reaction. The formed polymer was filtered and dried in vacuum.

### Kinetics of *rac*-LA polymerization

To determine the kinetics of the polymerization of *rac*-LA, a polymerization reaction in small scale at a temperature of 140 °C under nitrogen atmosphere were carried out. At 200:1 ratio the polymerizations were performed by charging 11.6 μmol of **1**–**4** (7 mg of **1**, 8 mg of **2**, 11 mg of **3**, and 8 mg of **4**) and 1.00 g of *rac*-LA (6.94 mmol). Aliquots were taken out at regular time intervals from the glass reactor under argon atmosphere and ^1^H NMR spectra were recorded to determine the % conversion of monomer into the corresponding polymer by comparing the methine proton of the unreacted monomer and polymer. Apparent rate constant (*k*_app_) were obtained from the slopes of the best fit lines from a plot of ln([*rac*-LA]_0_/[*rac*-LA]_*t*_) vs. time.

### Characterization of polymers

Molecular weights (*M*_n_) and molecular weight distributions (MWDs) of the polymer samples produced by the ROP of *rac*-LA were determined using gel permeation chromatography (GPC). GPC was performed on a Viscothek GPCmax instrument using a PFG column from PSS (Mainz, Germany, 300 mm × 8 mm, 5 μm particle size). The samples were eluted with DMF containing 5 mM LiBr as the mobile phase at a flow rate of 0.75 cm^3^ min^−1^ at 60 °C. The molecular weights were calculated relative to polystyrene standards from PSS using a conventional calibration of the refractive index detector. The samples were filtered through a nylon microfilter (0.2 μm) prior to the measurement.

## Electronic supplementary material

Below is the link to the electronic supplementary material.

Supplementary material 1 (DOCX 882 kb)
