# Supplementary material for: Copper(II) complexes with imino phenoxide ligands: synthesis, characterization, and their application as catalysts for the ring-opening polymerization of rac-lactide
Source: Monatsh Chem. 2016 Sep 5;147(11):1883–92. doi: 10.1007/s00706-016-1830-7 (PMC5063905; doi:10.1007/s00706-016-1830-7)
Supplement: Supplementary file 1 — Supplementary material 1 (DOCX 882 kb) [file 706_2016_1830_MOESM1_ESM.docx]

**Supporting Information**

**Copper(II) complexes with iminophenoxide ligands: Synthesis, characterization, and their application as catalysts for the ring opening polymerization of *rac*-lactide**

Mrinmay Mandal^1,2^, Kerstin Oppelt^1^, Manuela List^3^, Ian Teasdale^4^, Debashis Chakraborty^5,*^, Uwe Monkowius^1,*^

^1^Institute of Inorganic Chemistry, Johannes Kepler University Linz, Altenbergerstr. 69, 4040 Linz, Austria. Fax: +43 732 2468 9681; Tel: +43 732 2468 8814; E-mail: uwe.monkowius@jku.at

^2^Department of Chemistry, Indian Institute of Technology Patna, Patna 800 013, Bihar, India

^3^Institute for Chemical Technology of Organic Materials, Johannes Kepler University Linz, Altenbergerstr. 69, 4040 Linz, Austria

^4^Institute of Polymer Chemistry, Johannes Kepler University Linz, Altenbergerstr. 69, 4040 Linz, Austria

^5^Department of Chemistry, Indian Institute of Technology Madras, Chennai-600 036, Tamil Nadu, India. E-mail: dchakraborty@iitm.ac.in

**
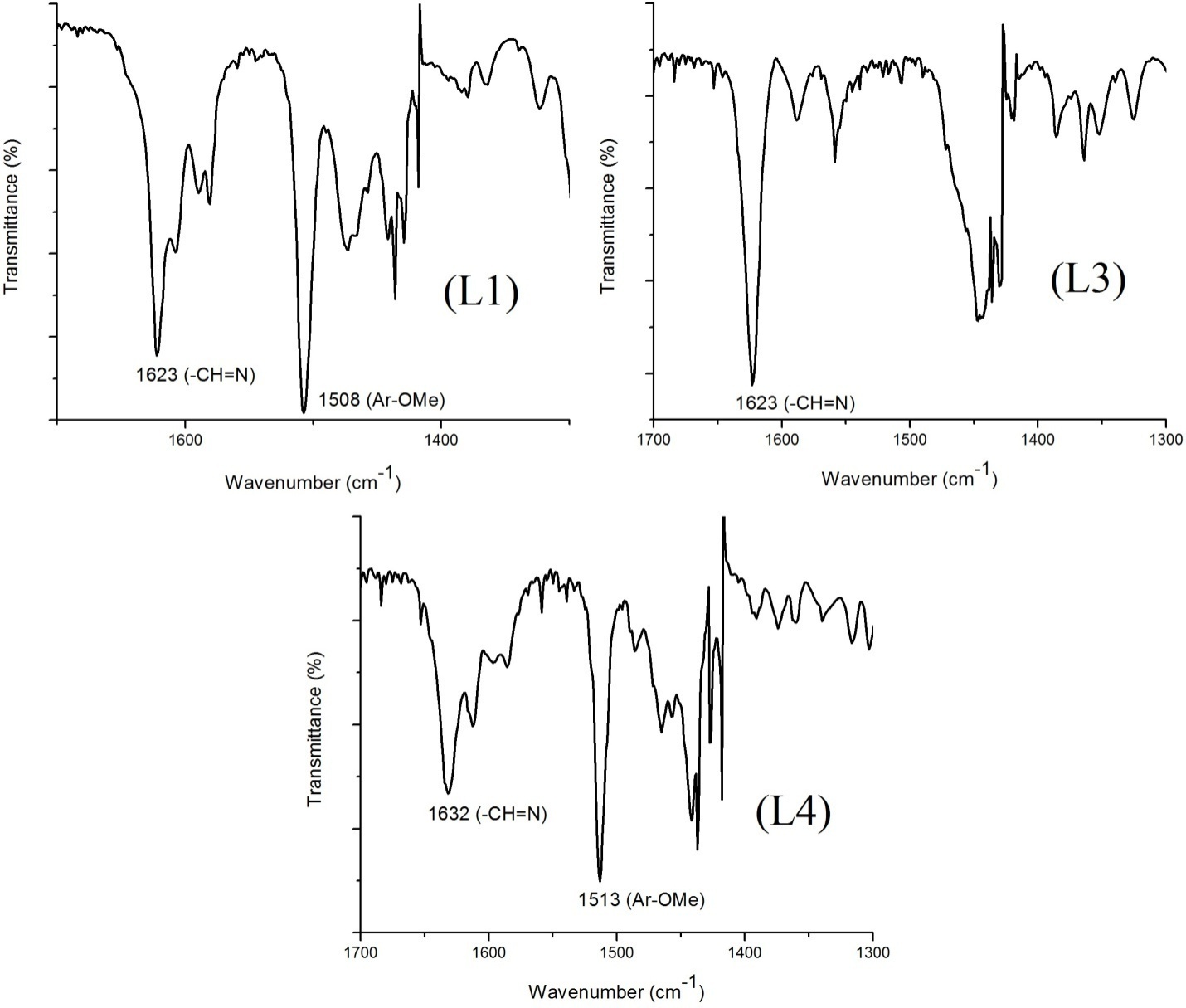
**

**Fig. S1.** IR spectra of the ligands (neat compound, ATR-mode)

**
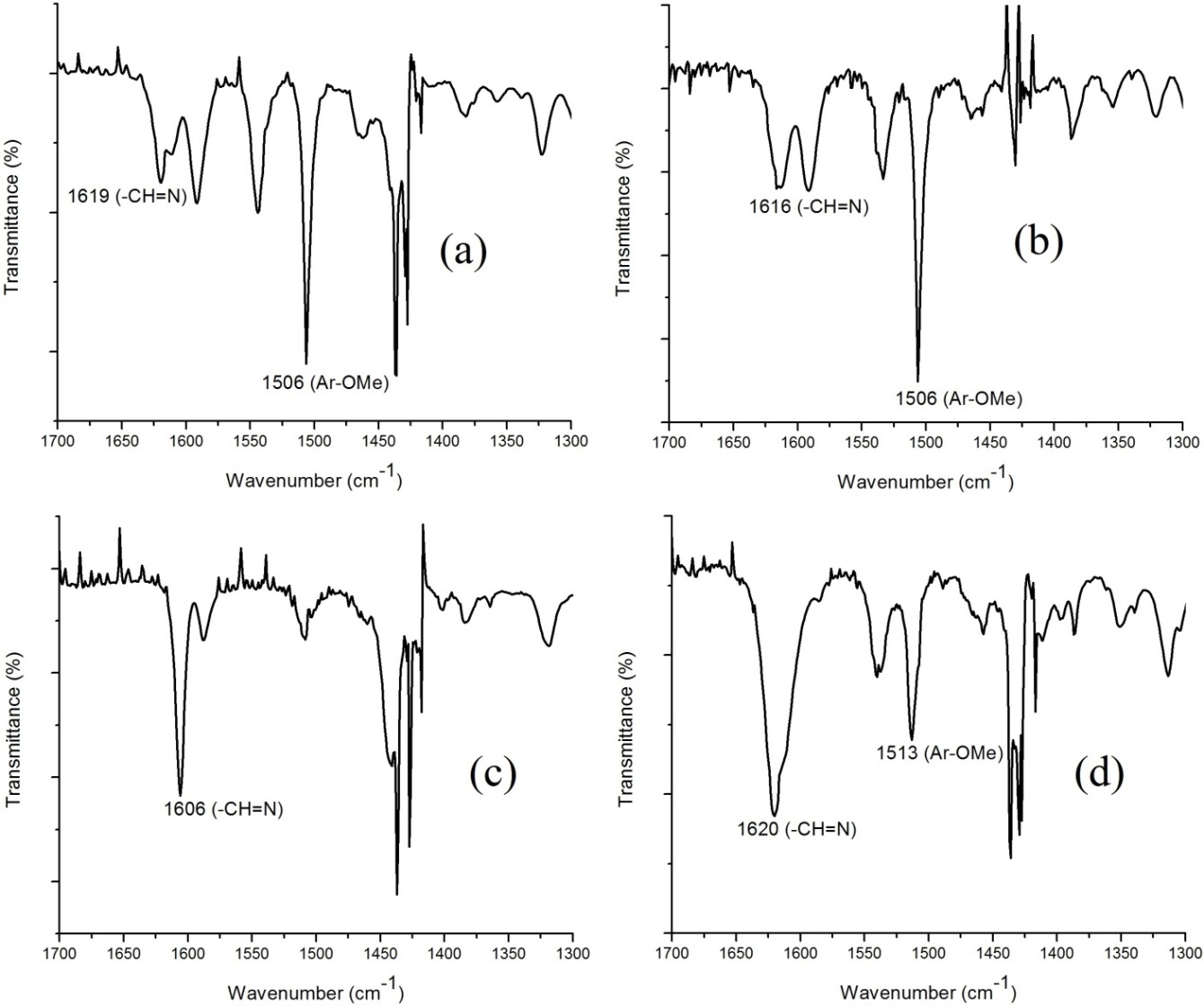
**

**Fig. S2.** IR spectra of compound **1** (a), **2** (b), **3** (c)and **4** (d) (neat complexes, ATR-mode).


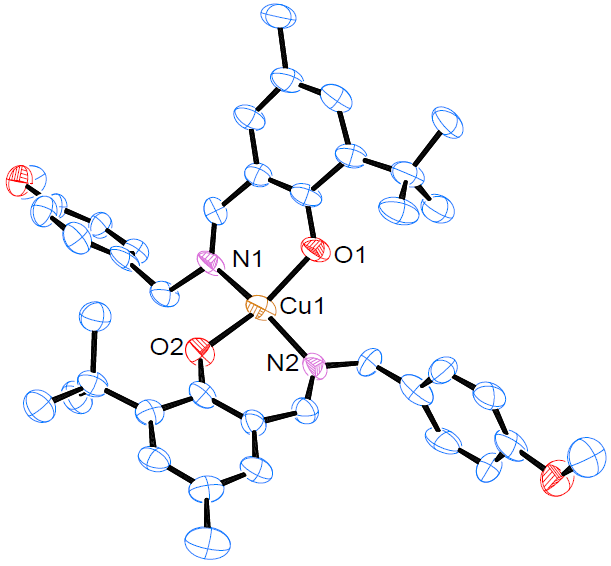


**Figure S3.** Molecular structure of **4**. Displacement ellipsoids were drawn at 50 % probability level. Hydrogen atoms are omitted for clarity.

**Table S1.** Crystal data for **4**.

| Compound | **4** |
| --- | --- |
| Empirical formula | C_40_H_48_CuN_2_O_4_ |
| Formula weight | 684.34 |
| Crystal system | triclinic |
| Space group | *P* 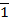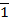 |
| Temp/K | 300 |
| *a* (Å) | 11.989(5) |
| *b* (Å) | 13.171(5) |
| *c* (Å) | 13.714(6) |
| *α* (°) | 84.208(12) |
| *β* (°) | 66.798(11) |
| *γ* (°) | 66.393(11) |
| *V* (Å^3^) | 1819.7(13) |
| *Z* | 2 |
| *D*_calc_ (g/cm^3^) | 1.249 |
| Reflns collected | 22176 |
| Indep. reflns | 5129 |
| Obs. reflns [I > 2σ(I)] | 3418 |
| Param. refin./restr. | 434/0 |
| Absorption correction | multi-scan |
| *R*_1_ | 0.098 |
| *wR_2_* | 0.308 |
| CCDC | 1448160 |

**Table S2** Selected bond lengths (Å) and bond angles (°) for **4**.

|  | **4** |
| --- | --- |
| Cu–O | 1.896(5)  1.897(5) |
| Cu–N | 1.947(7)  1.983(7) |
| O–Cu–O | 155.2(2) |
| N–Cu–N | 158.8(3) |
| O–Cu–N*  O–Cu–N | 92.2(2)/92.3(2)  93.5(2)/91.0(2) |


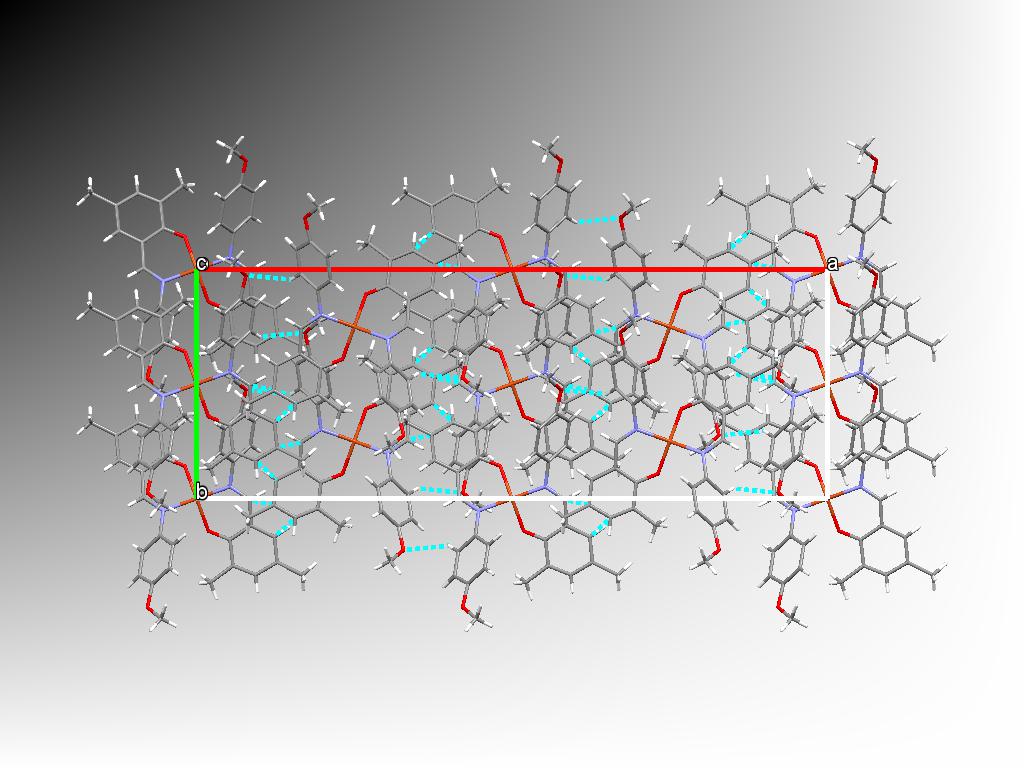


**Fig. S4.** Crystal packing diagram along *c*axis displaying CH/π, π/π, CH/O and C(benzylic)/πinteraction in complex**1**.


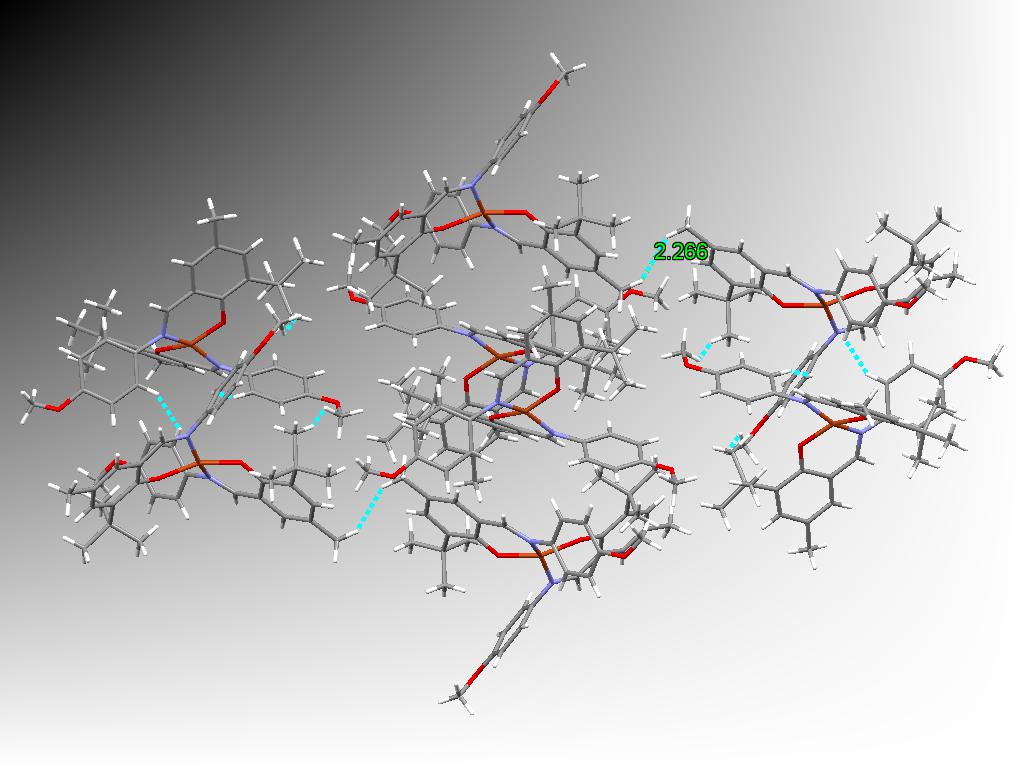


(a)


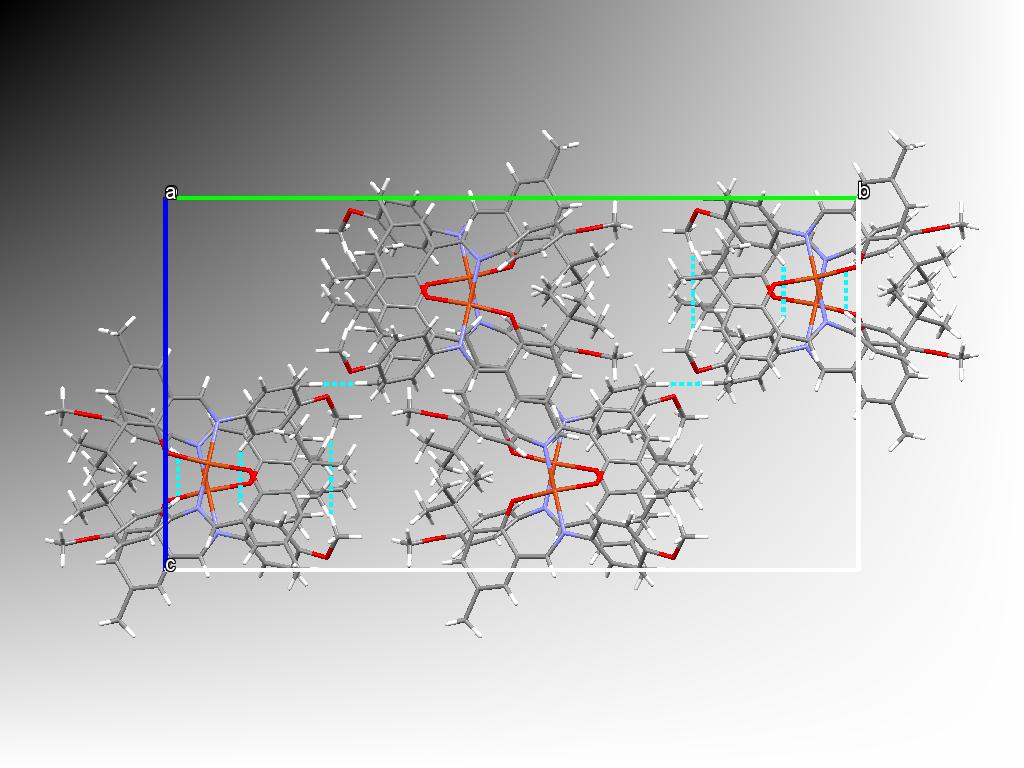


(b)

**Fig. S5.** Complex **2**: (a) CH/HC interaction (b) crystal packing diagramalong *a*axis.


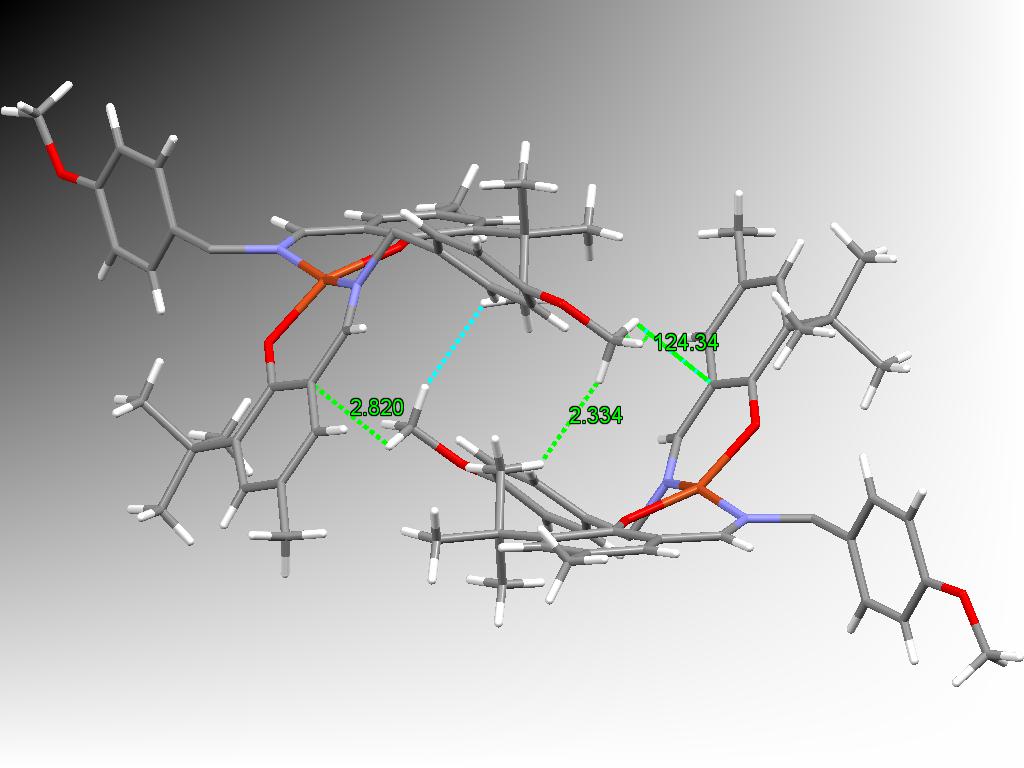


(a)


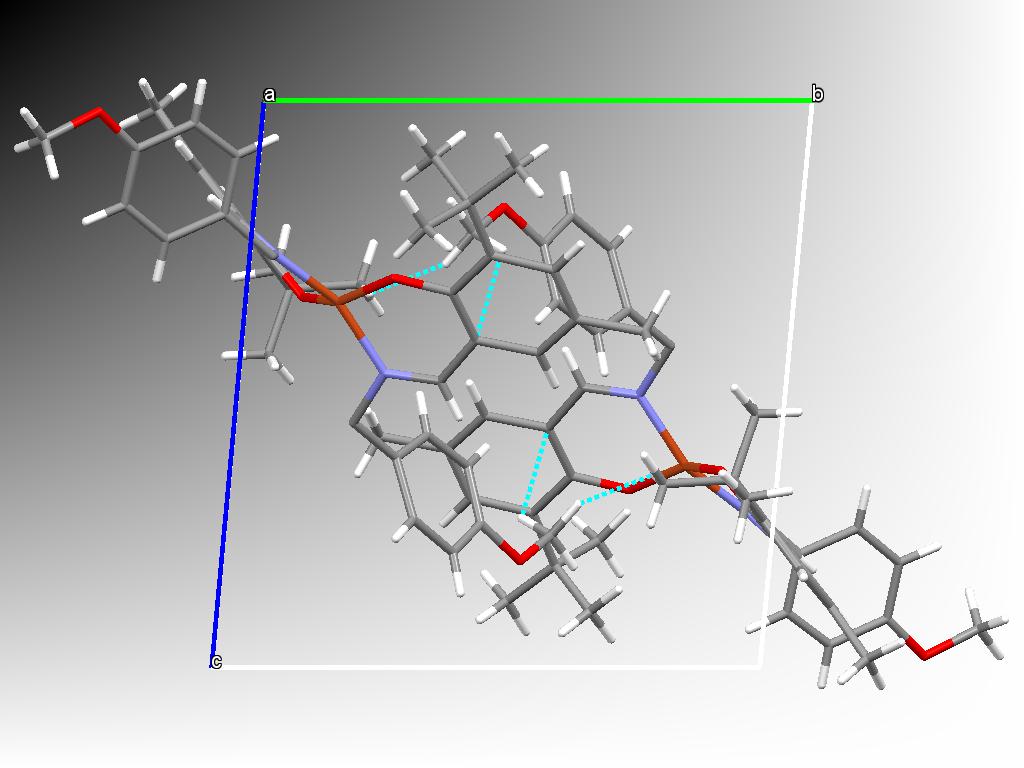


(b)

**Fig. S6.** (a) CH/π and CH/HC interaction in complex **4**. (b) Crystal packing diagram along *a*axis.

**Figure S7.** UV-VIS Absorption spectra of **1** – **4** in dichloromethane at high concentrations.
